# The Relationship Between Dentofacial and Body Postural Asymmetries in Patients with Malocclusions—A Cross-Sectional Clinical Study

**DOI:** 10.3390/medicina62040626

**Published:** 2026-03-25

**Authors:** Alexandra-Nina Botezatu, Eduard Radu Cernei, Elena Mihaela Cărăușu, Daniela Anistoroaei, Georgeta Zegan

**Affiliations:** 1 Department of Oral and Maxillofacial Surgery, Faculty of Dental Medicine, Grigore T. Popa University of Medicine and Pharmacy Iasi, 16 Universitatii Street, 700115 Iasi, Romania; alexandra.botezatu@yahoo.com (A.-N.B.); mihaelacarausu@yahoo.com (E.M.C.); anistoroaei_daniela@yahoo.com (D.A.); georgetazegan@yahoo.com (G.Z.); 2 Orthodontics Private Practice, 700056 Iasi, Romania

**Keywords:** facial asymmetry, body posture, malocclusions, angle classification, occlusal plane, dental midline, head posture

## Abstract

*Background and Objectives*: Dentofacial asymmetries are common in patients with malocclusions, while mild body postural asymmetries are frequently reported in otherwise healthy individuals. However, their interrelationship remains insufficiently investigated in adults without diagnosed spinal disorders. This study aimed to evaluate the association between dentofacial and body postural asymmetries in adults with malocclusions. *Materials and Methods*: A clinical cross-sectional observational study was conducted on 102 adults (18–45 years) with malocclusions and no spinal pathology. Standardized clinical morphometric examinations assessed dentofacial asymmetries (horizontal and vertical planes), dental parameters (dental midlines deviation and occlusal plane inclination), and body postural asymmetries (head, shoulder, trunk, pelvic, and lower limb alignment). Asymmetries were recorded using predefined clinical thresholds. Statistical analyses included the Wilcoxon signed-rank test, Pearson chi-square test, and Spearman’s rank correlation coefficient. *Results*: Dentofacial asymmetries were identified in both planes and occurred more frequently on the left side. Horizontal facial asymmetries were most common at the cheek (74.5%), nostril (66.7%), and mandibular angle levels (57.9%), and were influenced by sex, age, facial growth pattern, and facial profile (*p* ≤ 0.05). Mandibular dental midline asymmetry was present in 55.8% of patients. Body postural asymmetries were also frequent, particularly unilateral (60.8%) or anterior (55.9%) head inclination and shoulder asymmetries (54.9%), with a predominance on the left side and associations with age, body mass index, and postural attitude (*p* ≤ 0.05). Correlations were identified among facial asymmetries and among body postural asymmetries (*p* ≤ 0.01), indicating a bilateral distribution pattern. Additionally, right-sided facial asymmetries showed significant positive associations with right-sided body postural asymmetries (ρ = 0.197–0.229; *p* ≤ 0.05). *Conclusions*: Dentofacial and body postural asymmetries have been identified in adults with malocclusions and presented side-specific associations regarding the patterns of asymmetry.

## 1. Introduction

Facial asymmetry has been extensively investigated, with numerous studies describing its presence in both apparently healthy individuals and patients with malocclusions, allowing differentiation between physiological and pathological patterns. In the physiologic model, research on healthy subjects without craniofacial disorders has shown that mild facial asymmetry is common, with some degree of right–left variation in facial size and shape, representing a normal anatomical variation rather than pathology [1,2,3]. Although facial symmetry is traditionally associated with attractiveness, several studies have demonstrated that aesthetically pleasing faces may present minor functional asymmetries without a reduction in perceived beauty [4,5,6,7]. In contrast, in the pathological model, facial asymmetry has been associated with all major types of dental and skeletal malocclusions and congenital, developmental or acquired dentofacial deformities, potentially leading to functional and aesthetic impairments of varying severity [8,9,10,11,12]. The clinical threshold for the transition from physiological to pathological asymmetry is still debatable. However, based on previous orthodontic and anthropometric studies, a variation of ≥2 mm between the two halves of the face has been adopted, indicating that deviations beyond this magnitude are clinically perceptible and may have functional relevance [10,13].

Optimal posture is the result of the alignment of body segments that provides maximum physiological and biomechanical efficiency, protecting the body from the effect of gravity, and is clinically characterized by symmetry in the frontal and transverse planes [14]. Body postural asymmetry is defined as a measurable deviation in shoulder, pelvic, or trunk alignment in the frontal plane during orthostatic posture, with a threshold of ≥10 mm difference between bilateral landmarks [15]. Body postural asymmetry has also been documented in both adults and children. Studies on apparently healthy individuals indicate that small asymmetries of body segments may be present in orthostatic posture and are often considered within physiological limits [16,17,18,19,20]. Other investigations have focused on postural asymmetry in patients with systemic, musculoskeletal, or dentofacial conditions [21,22,23]. More recent research has emphasized the relationship between postural deviations, chronic musculoskeletal pain, and biomechanical interactions within the locomotor system, highlighting the complexity of postural control mechanisms [24,25,26,27].

The relationship between dentofacial asymmetry and postural deviations expressed as body asymmetries in apparently healthy individuals remains insufficiently explored, particularly in adults with malocclusions, and the existing findings are inconsistent. Early investigations primarily focused on patients with scoliosis, a structural three-dimensional spinal deformity associated with clinically evident body asymmetries. These studies suggested a possible association between dentofacial asymmetry and postural deviations, and between dental occlusion and scoliosis, raising the hypothesis that similar relationships may exist in non-structural, functional postural alterations [28,29,30,31]. Additional insights have been provided by studies investigating the interrelationships between dental occlusion and posture [32,33,34]. However, data addressing the association between dentofacial asymmetry without skeletal involvement and functional postural deviations are still lacking. Therefore, the aim of the present study was to investigate the relationship between dentofacial asymmetries and body postural asymmetries in patients with malocclusions and without diagnosed spinal disorders. This study hypothesizes that dentofacial asymmetries in patients with malocclusions may exhibit side-specific associations with functional body postural asymmetries, potentially reflecting biomechanical and neuromuscular interconnections within the craniofacial–postural system.

## 2. Materials and Methods

### 2.1. Study Design and Setting

A clinical cross-sectional observational study was conducted in a private orthodontic practice in Iasi, Romania, between January and March 2024. The study protocol comprised three consecutive stages: (1) clinical assessment of dentofacial measurements; (2) clinical assessment of body measurements; and (3) identification of associations between dentofacial and body postural asymmetries.

### 2.2. Patient Selection

The study sample included 102 patients with malocclusions and no diagnosed spinal disorders. Participants were recruited consecutively among patients attending for orthodontic consultation. As this represents a single-site convenience clinical sample, potential selection bias and limited external validity must be considered when interpreting the findings.

Inclusion criteria were as follows: age between 18 and 45 years, presence of malocclusions, absence of previous orthodontic treatment or permanent tooth extractions, and provision of written informed consent to participate in the study.

Exclusion criteria included patients undergoing active orthodontic treatment, presence of genetic or endocrine syndromes, surgically treated cleft lip and/or palate, diagnosed spinal disorders, age under 18 years or over 45 years, history of maxillofacial or orthopedic trauma or surgery, and failure to provide informed consent. Exclusion of spinal pathology was based on (1) medical history review, (2) patient self-report of prior orthopedic or neurological diagnoses, and (3) clinical postural screening during examination. Imaging or specialist orthopedic evaluation was not performed; therefore, undiagnosed or subclinical spinal deformities cannot be fully excluded.

### 2.3. Facial Clinical Examination

Facial examination was performed with patients standing upright, in a relaxed posture, with the head positioned naturally, eyes oriented forward, teeth in habitual occlusion, and lips relaxed, in accordance with previously described protocols [6]. Patients with long hair were instructed to tie it back, and all facial makeup was removed prior to examination to ensure accurate identification of cutaneous landmarks.

Using a dermatographic pencil, median and bilateral facial cutaneous reference points were marked according to the Farkas anthropometric system [35]. The following landmarks were identified:

- **Glabella cutaneon (G)**: the most prominent median point between the eyebrows;
- **Superciliare (SCI)**: the highest point on the superior margin of the medial eyebrow;
- **Exocanthion (EX)**: the point at the lateral commissure of the palpebral fissure;
- **Endocanthion (EN)**: the point at the medial commissure of the palpebral fissure;
- **Zygion cutaneon (ZY)**: the most lateral point of the zygomatic arch;
- **Pronasale (PRN)**: the most protruded point of the nasal apex;
- **Alare (AL)**: the most lateral point on each alar contour;
- **Cheilion (CH)**: the point at each labial commissure;
- **Gonion cutaneon (GO)**: the most lateral point at the mandibular angle;
- **Gnathion cutaneon (GN)**: the most inferior median point on the mandibular border.

The facial midsagittal line (MSL) was established using a plumb line perpendicular to the ground passing through point G [10]. Distances from the facial MSL to bilateral and median reference points were measured using an electronic digital caliper.

In the horizontal plane, a difference of ≤2 mm between right and left measurements was considered normal. Horizontal facial asymmetry was diagnosed when the difference exceeded 2 mm [10,13]. Median points (PRN and GN) were expected to coincide with the facial MSL; deviation indicated nasal or chin deviation to the right or left.

In the vertical plane, bilateral landmarks were considered symmetrical when no measurable vertical difference was detected relative to the reference horizontal line. Vertical facial asymmetry was diagnosed when one landmark was positioned inferiorly relative to its contralateral counterpart. After completion of the measurements, all facial markings were removed.

### 2.4. Dental Clinical Examination

Dental examination was conducted with the patient in the same standing position used for facial examination. A plastic cheek retractor was used to maintain adequate mouth opening. The maxillary and mandibular dental midlines were evaluated in maximum intercuspation [36].

Distances from the maxillary and mandibular dental midlines to the facial MSL were measured. A deviation of ≤2 mm was considered normal. Deviations greater than 2 mm were recorded as maxillary or mandibular dental asymmetry [37,38].

Occlusal plan inclination was assessed by asking patients to bite on a disposable wooden spatula positioned at the level of the permanent first molars. Under normal conditions, the spatula is horizontal. Occlusal plane asymmetry was diagnosed when a downward inclination toward the right or left side was observed [39].

### 2.5. Recording of Dentofacial Asymmetries

All dentofacial measurements were recorded using a custom-designed clinical examination form. This form included three sections: (1) horizontal and vertical measurements; (2) sociodemographic data (name, sex, age); and (3) orthodontic characteristics, including temporomandibular joint signs, facial growth pattern, facial profile, lower facial third proportion, and Angle classification.

### 2.6. Clinical Examination of the Head and Body

Body examination was performed with patients standing barefoot, wearing close-fitting clothing, with feet together, arms alongside the body, and gaze directed forward. Cutaneous body reference points were marked using self-adhesive labels placed over the clothing [40,41]. The following landmarks were identified:

- Tragion (T): superior depression of the tragus of the ear;
- Cervicale (C): cutaneous projection of the spinous process of the seventh cervical vertebra (C7);
- Acromiale (A): most lateral point of the acromion;
- Subscapulare (SS): inferior angle of the scapula;
- Iliocristale (IC): highest point of the iliac crest;
- Trochanterion (TR): superior-most point of the greater trochanter of the femur;
- Medial tibiale (TI): superior-most point of the medial tibial condyle;
- Tibial sphyrion (SPH): inferior-most point of the medial malleolus;

For posterior examination, the body MSL was established using a plumb line passing midway between the heels and extending vertically through the pelvis, spine, and cranium [42]. Distances from the body MSL to bilateral landmarks were measured using an electronic digital caliper.

In the horizontal plane, a difference of ≤10 mm between right and left measurements was considered normal. Horizontal body asymmetry was diagnosed when this threshold was exceeded [15].

In the vertical plane, body landmarks were considered symmetrical when no measurable vertical difference was detected relative to the reference horizontal line. Vertical body asymmetry was diagnosed when one landmark was positioned lower than its contralateral counterpart.

For body profile examination, a vertical reference line was established using a plumb line passing through the lateral malleolus and calcaneocuboid joint. Under normal conditions, this line passes through points TR, A, and T [42,43].

Unilateral head inclination relative to the body MSL was recorded as right or left head tilt [44]. An anterior displacement of point T relative to the body profile reference line was diagnosed as forward head posture [42,43].

### 2.7. Recording of Body Asymmetries

All body measurements were recorded in a custom-designed body asymmetry examination form, including: (1) horizontal and vertical measurements from posterior and profile views; (2) sociodemographic data; (3) anthropometric data (weight, height); and (4) body postural attitude.

Body mass index (BMI) was calculated using the formula BMI = weight (kg)/height^2^ (m^2^). Patients were classified as underweight (BMI < 18.5), normal weight (BMI 18.5–24.9), overweight (BMI 25.0–29.9) or obese (BMI ≥ 30) [45].

Postural attitude was considered optimal when head and trunk alignment was preserved. In the “standard posture” described by Kendall et al. (2005), the vertical thread passes through the external auditory canal, midway down the shoulder, slightly posterior to the center of the hip joint, slightly anterior to the axis of the knee joint, and slightly anterior to the lateral malleolus of the ankle [42]. When the vertical thread passes through the anterior third of the shoulder, an increased thoracic curvature associated with forward head posture occurs, being classified as kyphotic posture. When the vertical line passes through the posterior third of the shoulder, an anterior pelvic translation associated with thoracic curvature accentuation occurs, being classified as lordotic posture, in the absence of pathological causes [46,47].

### 2.8. Measurement Reliability and Examiner Calibration

All measurements were performed by a single examiner with clinical experience in orthodontic assessment. Prior to the study, the examiner underwent calibration sessions for landmark identification and measurement procedures. To assess intra-examiner reliability, measurements were repeated in a randomly selected subset of participants (10%) after a one-week interval under the same conditions. Method error was calculated using Dahlberg’s formula, and intra-class correlation coefficients (ICC) were computed to evaluate reliability. Facial and body measurements were recorded in separate stages to reduce observational bias.

### 2.9. Ethical Considerations

The study protocol was reviewed and approved by the Research Ethics Committee of the Grigore T. Popa University of Medicine and Pharmacy, Iasi, Romania (Approval No. 149/03.02.2022). All participants provided written informed consent prior to enrolment. No material, financial incentives were offered to the participants.

### 2.10. Statistical Analysis

All data were entered into a Microsoft Excel database and subsequently analyzed using SPSS software, version 27.0 (SPSS Inc., Chicago, IL, USA) for Windows.

For dentofacial and body distance measurements, descriptive statistical parameters were calculated, including arithmetic mean, standard deviation (SD), standard error of the mean (SEM), and median.

Paired right–left quantitative measurements were compared using the nonparametric Wilcoxon signed-rank test, as the data did not follow a normal distribution; therefore, Student’s *t* test was not applied. A *p* value ≤ 0.05 was considered statistically significant.

Comparative analyses of patients with facial and body asymmetries were performed according to sociodemographic, orthodontic, anthropometric, and postural variables, using the Pearson chi-square (χ^2^) test. Statistical significance was set at *p* ≤ 0.05.

The Spearman rank correlation coefficient (ρ) was used to assess associations between facial asymmetries and body postural asymmetries, as the variables were ordinal. Correlations were interpreted as positive when ρ > 0, negative when ρ < 0, and negligible when ρ ≈ 0. Correlation strength was classified as weak (0.1 ≤ ρ < 0.3), moderate (0.3 ≤ ρ < 0.5), or strong (ρ ≥ 0.5). Statistical significance was set at *p* ≤ 0.05.

Because this study was designed as an exploratory clinical investigation and multiple statistical comparisons were performed, no formal correction for multiple testing was applied. Therefore, statistically significant findings should be interpreted cautiously, and the reported associations should be considered hypothesis-generating.

Multivariable modeling was considered to control for potential confounders such as age, sex, body mass index, and postural characteristics. However, due to the moderate sample size and the limited number of observations within several variable categories, robust multivariable analyses were not performed to avoid statistical overfitting.

## 3. Results

### 3.1. Sample Characteristics

A total of 102 patients aged between 18 and 45 years (mean age 28.71 ± 9.06 years, SD) were investigated in this study, including 35 men (34.3%) with a mean age of 29.34 ± 9.56 years (SD) and 67 women (65.7%) with a mean age of 28.37 ± 8.84 years (SD).

Orthodontic data extracted from the clinical records showed that most patients did not present pathological signs of the temporomandibular joints (80.4%) and exhibited a normodivergent facial growth pattern (56.9%), a convex facial profile (88.2%), and equal thirds facial (62.7%) (Table 1).

The mean height of male patients was 177.74 ± 6.95 cm (SD), while that of female patients was 166.25 ± 6.29 cm (SD). The mean weight was 77.74 ± 13.91 kg (SD) in men and 59.66 ± 11.34 kg (SD) in women. Most patients presented kyphotic postural attitudes (59.8%), followed by lordotic postural attitudes (22.5%), while the fewest exhibited optimal postural attitudes (17.6%).

### 3.2. Evaluation of Dentofacial Measurements

The measured dentofacial distance values were evaluated to establish the diagnosis of facial asymmetry patients in the horizontal and vertical planes.

Application of the Wilcoxon test indicated highly statistically significant differences between right–left horizontal distance values measured from the SCI, EX, and EN points to the facial MSL (*p* = 0.001), which means that asymmetries are found only at the level of the eyebrows and eyes in the horizontal plane, in the upper third facial. No statistically significant differences were found between right–left distance values measured from the ZY, AL, CH, and GO points to the facial MSL (*p* > 0.05) (Table 2).

To identify patients with right–left facial asymmetries in the horizontal plane, differences between right- and left-side measurements of the bilateral landmarks’ SCI, EX, EN, ZY, AL, CH, and GO relative to the facial MSL were calculated. To identify patients with nasal and chin deviation to the right or left, distances from the median landmarks PRN and GN to the facial MSL were measured. Based on these calculations, patients presenting right- or left-sided facial asymmetries in the horizontal plane were established for this patient sample. No patients of horizontal asymmetry were identified at the level of the internal canthus or the left external canthus (Table 3).

By summing right- and left-sided facial asymmetry patients in the horizontal plane, the frequencies of asymmetry for the examined facial elements in this patient sample were identified (Table 3).

By comparing the positions of the bilateral points SCI, EX, EN, ZY, AL, CH, and GO on the right and left sides of the face, patients with right/left facial asymmetries in the vertical plane were diagnosed. No patients of vertical asymmetry were identified at the level of the right ex point or the left EN point (Table 4).

By summing the right- and left-sided facial asymmetry patients in the vertical plane, the frequencies of asymmetries of the examined facial elements were identified (Table 4).

To identify patients with asymmetries of the maxillary and mandibular dental midlines in the horizontal plane, differences between the measured distances from the dental midlines to the facial MSL were calculated. Downward unilateral inclination of the occlusal plane was recorded in the patient chart. Based on these calculations and data, the frequencies of patients with right/left dental midline asymmetries in the horizontal plane and the frequencies of patients with unilateral right/left occlusal plane inclination in the vertical plane were established (Table 5).

Patients presented one or more facial asymmetries on the right and/or left side, in the horizontal and/or vertical plane, in accordance with the clinical records.

By combining paired facial asymmetries identified in both planes, global facial asymmetries were established. Global asymmetries were present on the right side in 50.0% of and on the left side in 59.8% of patients (Table 6)

Paired and global facial asymmetries were analyzed in relation to patients’ sociodemographic and orthodontic characteristics (Table 7 and Table 8). Comparisons of patients with facial asymmetries according to the size of the lower face could not be interpreted, because some categories had an insufficient number of cases. No statistically significant differences were found in patients with facial asymmetries according to temporomandibular joint signs or Angle classification of malocclusions (*p* > 0.05).

### 3.3. Evaluation of Body Measurement

To establish the diagnosis of patients presenting with horizontal and vertical body asymmetries, the values of distances measured at the body level were assessed.

Application of the Wilcoxon signed-rank test revealed highly statistically significant differences between right–left distance values in the horizontal plane, measured from the body MSL to points IC (*p* = 0.001) and TR (*p* = 0.007), which means that asymmetries are found only at the level of the pelvis and ankles in the horizontal plane, in the lower segments of the body. No statistically significant differences were identified between the distances measured from the body MSL to points A, SS, TI, and SPH (Table 9).

Based on the calculated right–left differences between the body MSL and the bilateral landmarks A, SS, IC, TR, TI, and SPH, the diagnosis of patients with paired right–left horizontal body asymmetries was established (Table 10).

Patients with right-sided and left-sided body asymmetries in the horizontal plane were aggregated, and the frequencies of asymmetries in the examined body elements were determined (Table 10).

The positions of the bilateral landmarks A, SS, IC, TR, TI, and SPH on the right and left sides of the body were compared to identify patients of right–left body asymmetries in the vertical plane. No vertical asymmetries were identified at the TI and SPH landmarks (Table 11).

Patients with right-sided and left-sided body asymmetries in the vertical plane were pooled, and the frequencies of asymmetries of the examined body elements were determined (Table 11).

Unilateral head tilt relative to the body MSL and anterior head inclination relative to the body profile reference line were recorded in the patient chart, and the frequencies of patients presenting head postural inclinations were determined (Table 12).

Patients exhibited one or more postural body asymmetries on the right and/or left side, in the horizontal and/or vertical planes, as recorded in the clinical charts after calculation of measurement differences.

Patients with paired right–left body asymmetries in the horizontal and vertical planes were further combined to identify patients with global body asymmetries (Table 13).

Patients with paired right–left body asymmetries in the horizontal and vertical planes, as well as patients with global body asymmetries, were compared with patients’ sociodemographic characteristics, BMI, and postural body attitudes (Table 14).

### 3.4. Identification of Correlations Between Facial and Body Postural Asymmetries

The application of Spearman’s rank correlation coefficient enabled the identification of associations between facial asymmetries and postural body asymmetries in the study group.

Highly significant negative and moderate correlations (*p* < 0.001) were identified between paired right-sided facial asymmetries in the horizontal plane and paired left-sided facial asymmetries in the horizontal plane (ρ = −0.538), and a weak to moderate correlation between global right-sided and global left-sided facial asymmetries (ρ = −0.431) (Table 15). These correlations reflect inverse proportional relationships between the respective asymmetry variables.

Highly significant positive and weak correlations (*p* < 0.001) were observed between paired right-sided body asymmetries in the horizontal plane and paired left-sided body asymmetries in the vertical plane (ρ = 0.288, *p* = 0.003), which indicates direct proportional relationships between the respective postural asymmetry patterns.

A highly statistically significant negative and weak correlation was identified between paired right-sided and paired left-sided facial asymmetries in the vertical plane (ρ = −0.337; *p* < 0.001) (Table 16), indicating an inverse proportional relationship between these variables.

Weak, positive, and statistically significant correlations were identified between paired right-sided facial asymmetries in the horizontal plane and paired right-sided body asymmetries in the vertical plane (ρ = 0.229, *p* = 0.021), and between global right-sided facial asymmetries and global right-sided body asymmetries (ρ = 0.197, *p* = 0.047) (Table 17). These findings suggest the presence of weak statistical associations between certain facial and postural asymmetry patterns in the examined patients. Given the cross-sectional design and the low magnitude of the correlation coefficients, these relationships should be interpreted cautiously and cannot be considered evidence of causal or mechanistic interactions.

## 4. Discussion

### 4.1. Dentofacial Asymmetries

In the present study, clinical examination and quantitative facial and dental measurements demonstrated the presence of dentofacial asymmetries in the investigated cohort. Comparative analysis of paired right–left facial distances in the horizontal plane revealed differences between the two hemifaces across multiple anatomical landmarks. Mean values were higher on the left side at the level of the eyebrows, eyes, and lip commissures, whereas greater mean values were observed on the right side at the level of the cheeks, nostrils, and mandibular angles. However, statistically significant differences were detected only at the level of the eyebrows and eyes, indicating that most facial asymmetries were mild and within a range considered clinically acceptable.

These findings support the concept that absolute facial symmetry is uncommon in the general population and represents an idealized anatomical construct rather than a physiological norm. Previous studies have consistently reported the presence of minor facial asymmetries in individuals without overt craniofacial pathology [3,6,48,49]. Moreover, the absence of a consistent pattern regarding side dominance in facial asymmetry has been highlighted in the literature. Several authors have reported larger dimensions on the right hemiface [9,35,50,51,52], whereas others observed a predominance of left-sided facial dimensions [5].

In the present sample, horizontal facial asymmetries defined in relation to the facial MSL were most frequently identified at the level of the cheeks, nostrils, and mandibular angles. This distribution is consistent with previous reports indicating that laterally positioned facial landmarks, such as zygion and gonion, exhibit greater variability and are more prone to asymmetry than centrally located structures [5,50,53].

The prevalence of malar asymmetry reported in the literature varies considerably, largely due to differences in study design, diagnostic criteria, and cut-off values. Wang et al. (2024) reported a relatively low prevalence of malar asymmetry (7%) in patients with skeletal Class III malocclusion when a 3 mm threshold was applied [54], whereas Moubayed et al. (2012) identified malar asymmetry in approximately 40% of asymptomatic young adults using a 2 mm cut-off [55]. Such methodological heterogeneity may explain discrepancies between published data and the results of the present study.

Regarding nasal asymmetry, the present study identified a low prevalence in comparison with studies conducted in surgical or rhinoplasty-oriented populations. Previous investigations focusing on patients seeking nasal or orthognathic surgery have reported markedly higher prevalences of nasal deviation, ranging from 46% to over 90% [56,57,58,59]. These differences emphasize the influence of sample selection and clinical context on the reported prevalence of facial asymmetries.

Low prevalences of asymmetry were also observed at the level of the lip commissures, eyebrows, and eyes. These findings agree with three-dimensional facial analyses demonstrating minimal asymmetry in the periocular and perioral regions in individuals without significant dentofacial deformities [1,60,61].

Vertical facial asymmetries, assessed by paired right–left comparisons in the vertical plane, were more frequently identified at the level of the eyebrows and eye commissures than in the horizontal plane. This observation is consistent with previous studies reporting that facial asymmetries are often more pronounced in the vertical dimension, possibly due to differences in vertical growth patterns and neuromuscular function [2,51].

Dental asymmetries were evaluated by analyzing the position of the maxillary and mandibular dental midlines relative to the facial MSL. In the present study, mandibular dental asymmetries were more prevalent than maxillary asymmetries, a finding that aligns with earlier clinical and epidemiological studies [62,63]. The lower prevalence of unilateral occlusal plane inclination observed in this cohort is also consistent with previous orthodontic investigations [8].

A notable finding of the present study was the predominance of paired facial asymmetries on the left side, in the horizontal plan. Importantly, only left-sided asymmetries demonstrated statistically significant associations with sex and selected orthodontic variables. While several studies have reported a higher prevalence or greater severity of facial asymmetry in males [2,64,65], other investigations failed to identify sex-related differences [50,66,67]. These discrepancies suggest that sex-related patterns of asymmetry may be population-dependent and influenced by ethnic, developmental, and methodological factors.

Patients with paired left-sided facial asymmetries in the vertical plane showed significant age-related differences, with a higher prevalence in individuals younger than 30 years. These findings contrast with Ferrario et al. (2001), who reported no association between age and facial asymmetry [50], and with Choi et al. (2016), who observed stable asymmetry direction across adulthood with only weak negative correlations with age [68]. In contrast, other studies have suggested that facial asymmetry may increase with age [69,70], highlighting the inconsistency of existing evidence.

Paired right-sided facial asymmetries in the horizontal plane were more prevalent in normodivergent growth patterns, whereas paired left-sided vertical asymmetries were more frequent in hypodivergent patients. These results are not fully consistent with previous findings, which either associated asymmetry with hyperdivergent patterns [71] or reported no significant relationship between facial asymmetry and vertical skeletal pattern [72,73].

Additionally, paired left-sided vertical facial asymmetries were more frequent in patients with a convex facial profile, a relationship that has been scarcely investigated. Duran et al. (2020) reported no association between facial profile convexity and the perception of facial asymmetry [74].

### 4.2. Body Postural Asymmetries

In the present cohort, clinical assessment and quantitative measurements revealed the presence of body asymmetries in both the horizontal and vertical planes. In the horizontal plane, mean values were higher on the right side at the level of the shoulders, scapulae, hips, and knees, whereas greater mean values were observed on the left side at the level of the pelvis and ankles. Statistically significant right–left differences were identified only for pelvic and ankle measurements, indicating that most body asymmetries were subtle and characterized by considerable interindividual variability. These findings are consistent with previous studies demonstrating that postural and body asymmetries are commonly observed even in healthy individuals and are often influenced by functional dominance, habitual posture, and musculoskeletal adaptations [16,20,75].

Analysis of paired body asymmetries in the horizontal plane revealed the highest prevalences for asymmetry, whereas asymmetries involving the lower limbs were less frequent. In the vertical plane, shoulder asymmetry was the most prevalent finding, while scapular, pelvic, and hip asymmetries occurred less frequently. Unilateral and anterior head tilt in posture showed the highest frequency compared with the prevalence of other postural body asymmetries. This pattern agrees with previous investigations highlighting the role of muscular imbalance, cervical posture, and spinal alignment in the development of postural asymmetries [20,76].

Like dentofacial findings, body asymmetries in the present study were more frequently observed on the left side. Only left-sided body asymmetries showed statistically significant associations with patient-related variables. Specifically, left-sided paired vertical body asymmetries were influenced by age, with higher prevalence observed in individuals older than 30 years. Additionally, left-sided paired horizontal and vertical body asymmetries were influenced by BMI, being more frequent in normal weight individuals. These associations may reflect cumulative functional loading, age-related musculoskeletal changes, or characteristics specific to the studied population.

The relationship between BMI and postural asymmetry remains controversial in the literature. Grivas et al. (2009) reported an association between low BMI and increased trunk asymmetry, whereas de Miranda et al. (2022) identified sagittal postural alterations in individuals with higher BMI [77,78]. Such inconsistencies underscore the multifactorial nature of postural asymmetry and the influence of confounding variables, including physical activity level and musculoskeletal health.

Furthermore, left-sided paired horizontal body was influenced by postural attitude, with a higher prevalence observed in individuals presenting a kyphotic posture. Comparable associations between trunk asymmetry and sagittal spinal curvatures have been reported in both pediatric and adult populations [79], supporting the notion that sagittal spinal alignment may influence the expression of asymmetry in the frontal and transverse planes.

### 4.3. Relationships Between Facial and Body Postural Asymmetries

Associations among facial asymmetries, among body postural asymmetries, and between facial and body postural asymmetries were demonstrated by identifying interdependent relationships, statistically significant, both positive and negative. Weak and moderate associations were observed between paired and global facial or body asymmetries affecting the opposite sides, in both planes, indicating a consistent bilateral distribution pattern within segmental facial or body asymmetries alone.

In contrast, associations between facial and body postural asymmetries were exclusively limited to the right side, in both horizontal and vertical planes. This suggests a more consistent pattern of correspondence between facial asymmetry and postural misalignment when considering craniofacial–body relationships. To the best of our knowledge, such interdependencies, identified through clinical measurements in patients with malocclusions, have not been previously reported.

Previous studies have yielded heterogeneous results. Zepa et al. (2003) found no influence of clinically assessed thoracic or lumbar asymmetry on facial asymmetry, although a predominance of right cervical tilt was observed [80]. Similarly, Arienti et al. (2017), in a large adolescent cohort, reported no direct association between facial and body asymmetry, despite correlations between jaw position and cervical posture [81]. In contrast, Shuncheng et al. (2013) demonstrated that greater mandibular deviation was associated with increased coronal spinal curvature in young adults, suggesting a structural cranio-spinal relationship [82]. Primozic et al. (2023) reported no strong correlation between overall back asymmetry and facial asymmetry in prepubertal subjects; however, asymmetry of the upper trunk was significantly greater in individuals with asymmetric faces, with the mandibular region being the most affected facial area [83]. More recently, Ono et al. (2025) identified associations between facial asymmetry and thoracic vertebral deviation in adult female patients, although the directionality of asymmetry was not assessed [11].

Overall, the present findings support the existence of specific, side-dependent relationships between facial and body postural asymmetries, partially aligning with previous evidence suggesting craniofacial–postural interconnections, while also contributing novel clinical data to this field. Importantly, the present results demonstrate statistical associations rather than causal relationships. Although the previous literature proposes biomechanical and neuromuscular interconnections between craniofacial structures and global posture, the cross-sectional design and clinical measurement approach used in this study do not permit the inference of mechanistic pathways.

Alternative explanations for the observed side-dependent patterns must be considered. These include variability in clinical landmark identification, examiner-related measurement error, habitual stance asymmetry and limb dominance, unmeasured musculoskeletal adaptations, visual or vestibular influences on head posture, and occupational or lifestyle-related postural habits. Such factors may influence both craniofacial and body alignment independently, potentially contributing to the associations observed.

It is also important to distinguish statistical significance from clinical significance. Although several associations reached statistical significance, most detected asymmetries were mild and within ranges previously reported in individuals without major craniofacial or spinal pathology. Similarly, the magnitude of the correlation coefficients was predominantly weak, suggesting that the observed relationships may reflect subtle morphological or postural variations rather than clinically meaningful functional interactions. Therefore, the results should be interpreted primarily as observational findings that may guide future hypothesis-driven research.

### 4.4. Study Limitations

Several limitations should be considered when interpreting the present findings. First, the study relied on direct clinical identification of cutaneous landmarks and plumb-line alignment rather than three-dimensional imaging or instrumented postural analysis systems. Dentofacial and body asymmetries were therefore assessed using two-dimensional clinical measurements, which may not fully capture complex three-dimensional structural differences. Additionally, the identification of surface landmarks and evaluation of vertical alignment may introduce measurement variability. Consequently, the accuracy of the measurements may be lower than that achievable with digital imaging or motion-analysis technologies.

Although intra-examiner calibration was performed, inter-examiner reliability was not assessed, and clinical measurements are inherently more operator-dependent than digital methods.

The study design was cross-sectional and exploratory. No correction for multiple statistical testing was applied despite numerous comparisons, increasing the risk of Type I error. Analyses were primarily bivariate, and multivariable modeling was not performed; therefore, potential confounding by age, sex, BMI, and postural characteristics cannot be excluded.

Exclusion of spinal pathology was based on medical history, self-report, and clinical screening. Imaging or specialist orthopedic evaluation was not performed, meaning subclinical or undiagnosed spinal deformities cannot be ruled out.

Future investigations may benefit from incorporating radiographic screening or specialist orthopedic evaluation to more reliably exclude structural spinal deformities and to further clarify potential craniofacial–postural relationships.

Additionally, the sample represents a single-site convenience clinical population from a private orthodontic practice, which limits external validity and generalizability.

Finally, the clinical morphometric approach does not capture three-dimensional differences in shape or volume between facial and body halves. For these reasons, the findings should be interpreted as preliminary and hypothesis-generating.

## 5. Conclusions

Dentofacial and body postural asymmetries were observed in this adult sample of patients with malocclusions, with most asymmetries being mild in magnitude. Facial asymmetries were more frequently identified in the horizontal plane at laterally positioned landmarks, while body postural asymmetries commonly involved head position and shoulder alignment. Weak statistical associations were observed among certain facial variables, among postural variables, and between selected facial and postural asymmetry patterns.

However, these findings reflect associations derived from a cross-sectional clinical assessment and do not establish causal or mechanistic relationships. The observed side-related patterns and subgroup differences should be interpreted cautiously, as they may be influenced by measurement variability, unmeasured confounders, and the characteristics of the single-site convenience sample.

Within these limits, the study contributes clinical observational data on the coexistence of dentofacial and non-structural postural asymmetries in orthodontic patients, but further research using validated measurement protocols, reliability assessment, and multivariable analytical models is required before drawing firm clinical implications.

These findings should therefore be interpreted as exploratory and hypothesis-generating, requiring confirmation through future studies using larger samples, multivariable analytical approaches, and three-dimensional measurement techniques.

## Figures and Tables

**Table 1:** Orthodontic characteristics of the patients.

Variable	Category	n	%
**Temporomandibular joints**	Asymmetrical condylar excursions	9	8.8
	Normal	82	80.4
	Joint sounds	11	10.8
**Facial growth pattern**	Hyperdivergent	11	10.8
	Hypodivergent	33	32.4
	Normodivergent	58	56.9
**Facial profile**	Concave	3	2.9
	Convex	90	88.2
	Straight	9	8.8
**Lower third facial**	Equal	64	62.7
	Decreased	37	36.3
	Increased	1	1.0
**Angle classification**	Class I	40	39.2
	Class II Division 1	5	4.9
	Class II Division 2	46	45.1
	Class III	11	10.8

**Table 2:** Facial distance measurements in the horizontal plane.

Distance	Side	Mean	SD	SEM	Minimum	Maximum	Median	Wilcoxon Z	*p*
**SCI–MSL**	Right	35.50	2.77	0.27	31.28	41.63	34.24	−3.176	0.001 **
	Left	35.80	2.62	0.25	31.86	41.99	34.99		
**EX–MSL**	Right	55.45	2.05	0.20	51.78	60.01	54.66	−4.310	<0.001 **
	Left	55.81	1.92	0.19	52.11	60.92	55.19		
**EN–MSL**	Right	17.94	0.85	0.08	16.17	19.96	17.98	−4.835	<0.001 **
	Left	18.26	0.84	0.08	16.21	19.95	18.16		
**ZY–MSL**	Right	87.67	9.47	0.93	78.11	103.98	83.12	−0.664	0.102
	Left	87.59	8.98	0.88	78.03	104.81	81.92		
**AL–MSL**	Right	34.05	1.89	0.18	30.16	38.07	34.01	−1.634	0.102
	Left	33.84	1.95	0.19	29.79	37.54	34.12		
**CH–MSL**	Right	31.98	1.43	0.14	27.93	34.21	32.27	−1.704	0.088
	Left	32.19	1.32	0.13	28.13	34.26	32.29		
**GO–MSL**	Right	102.60	5.26	0.52	96.95	111.17	99.89	−0.217	0.828
	Left	102.58	4.91	0.48	95.32	111.65	100.16		

Wilcoxon signed-rank test. ** Highly statistically significant when *p* ≤ 0.01.

**Table 3:** Patients with right–left facial asymmetries in the horizontal plane.

Distance	Asymmetry	n	%
**SCI–MSL**	Right	1	1.0
	Left	2	2.0
	No asymmetry	99	97.1
**EX–MSL**	Right	1	1.0
	No asymmetry	101	99.0
**EN–MSL**	No asymmetry	102	100.0
**ZY–MSL**	Right	32	31.4
	Left	44	43.1
	No asymmetry	26	25.5
**PRN–MSL**	Right	14	13.7
	Left	6	5.9
	No asymmetry	82	80.4
**AL–MSL**	Right	26	25.5
	Left	42	41.2
	No asymmetry	34	33.3
**CH–MSL**	Right	10	9.8
	Left	9	8.8
	No asymmetry	83	81.4
**GO–MSL**	Right	28	27.5
	Left	31	30.4
	No asymmetry	43	42.2
**GN–MSL**	Right	6	5.9
	Left	2	2.0
	No asymmetry	94	92.2

**Table 4:** Patients with right–left facial asymmetries in the vertical plane.

Point	Asymmetry	n (%)
**SCI**	Right	1 (1.0)
	Left	9 (8.8)
	No asymmetry	92 (90.2)
**EX**	Left	3 (2.9)
	No asymmetry	99 (97.1)
**EN**	Right	3 (2.9)
	No asymmetry	99 (97.1)
**ZY**	Right	1 (1.0)
	Left	1 (1.0)
	No asymmetry	100 (98.0)
**AL**	Right	1 (1.0)
	Left	2 (2.0)
	No asymmetry	99 (97.1)
**CH**	Right	2 (2.0)
	Left	3 (2.9)
	No asymmetry	97 (95.1)
**GO**	Right	1 (1.0)
	Left	3 (2.9)
	No asymmetry	98 (96.1)

**Table 5:** Patients with dental asymmetries in the horizontal and vertical planes.

Plane	Measurement	Asymmetry/Inclination	n (%)
**Horizontal**	Maxillary dental midline–MSL	Right	15 (14.7)
		Left	7 (6.9)
		No asymmetry	80 (78.4)
	Mandibular dental midline–MSL	Right	23 (22.5)
		Left	34 (33.3)
		No asymmetry	45 (44.1)
**Vertical**	Occlusal plane inclination	Right	3 (2.9)
		Left	3 (2.9)
		No inclination	96 (94.1)

**Table 6:** Patients with paired right–left and global facial asymmetries.

Type of Asymmetry	Status	Right Side, n (%)	Left Side, n (%)
**Paired facial asymmetries (horizontal plane)**	Absent	54 (52.9)	47 (46.1)
	Present	48 (41.1)	55 (53.9)
**Paired facial asymmetries (vertical plane)**	Absent	97 (95.1)	89 (87.3)
	Present	5 (4.9)	13 (12.7)
**Global facial asymmetries**	Absent	52 (51.0)	41 (40.2)
	Present	51 (50.0)	61 (59.8)

**Table 7:** Association between facial asymmetries and gender (Pearson’s chi-square test).

Type of Dentofacial Asymmetry	Status	Men, n (%)	Women, n (%)	χ^2^	*p* Value
**Paired facial asymmetries (left side, horizontal plane)**	Absent	9 (25.7)	38 (56.7)	12.475	0.029 *
	Present	26 (74.3)	29 (43.3)		
		**18–45 years, n (%)**	**Over 30 years, n (%)**		
**Paired facial asymmetries (left side, vertical plane)**	Absent	51 (82.3)	38 (95.0)	7.852	0.049 *
	Present	11 (17.7)	2 (5.0)		

Values are presented as *n* (% within gender). Pearson’s chi-square test (χ^2^). * Statistically significant at *p* ≤ 0.05.

**Table 8:** Associations between left-sided paired and global facial asymmetries and orthodontic characteristics (Pearson’s chi-square test).

Facial Asymmetry Type	Orthodontic Characteristic	Category	Absent, n (%)	Present, n (%)	χ^2^	*p* Value
**Paired asymmetries (right side, horizontal plane)**	Facial growth pattern	Hyperdivergent	3 (27.3)	8 (72.7)	21.501	0.044 *
		Hypodivergent	16 (48.5)	17 (51.5)		
		Normodivergent	35 (63.6)	23 (36.4)		
**Paired asymmetries (left side, vertical plane)**	Facial growth pattern	Hyperdivergent	10 (90.9)	1 (9.1)	13.093	0.042 *
		Hypodivergent	26 (78.8)	7 (21.2)		
		Normodivergent	53 (91.4)	5 (8.5)		
**Paired asymmetries (left side, vertical plane)**	Facial profile	Concave	3 (100.0)	0 (0.0)	13.974	0.030 *
		Convex	79 (87.8)	11 (12.2)		
		Straight	7 (77.8)	2 (22.2)		

Values are presented as *n* (% within category). Pearson’s chi-square test (χ^2^). * Statistically significant at *p* ≤ 0.05.

**Table 9:** Measurements of body distances in the horizontal plane.

Distance	Side	Mean	SD	SEM	Minimum	Maximum	Median	Wilcoxon Z	*p*
**MSL–A**	Right	206.54	12.47	1.23	187.49	239.43	199.48	−0.192	0.848
	Left	205.84	13.05	1.29	163.27	240.54	201.64		
**MSL–SS**	Right	92.54	3.54	0.35	84.46	99.54	92.72	−0.769	0.442
	Left	92.46	3.85	0.38	85.03	101.21	91.76		
**MSL–IC**	Right	180.68	19.17	1.89	157.65	223.19	167.53	−4.151	<0.001 **
	Left	182.52	19.51	1.93	160.17	223.89	174.17		
**MSL–TR**	Right	196.68	16.24	1.60	179.32	240.98	189.28	−2.699	0.007 **
	Left	195.70	24.15	2.39	22.98	241.38	188.35		
**MSL–TI**	Right	3.03	10.74	1.06	0.00	52.00	0.00	−1.609	0.108
	Left	1.69	6.30	0.62	0.00	31.00	0.00		
**MSL–SPH**	Right	1.83	8.45	0.83	0.00	54.00	0.00	−0.734	0.463
	Left	2.04	8.88	0.88	0.00	56.00	0.00		

Wilcoxon signed-rank test. ** Highly statistically significant when *p* ≤ 0.01.

**Table 10:** Distribution of right–left body asymmetries in the horizontal plane.

Parameter	Asymmetry Direction	n (%)
**MSL–A**	Right asymmetry	21 (20.6)
	Left asymmetry	30 (29.4)
	Symmetric	51 (50.0)
**MSL–SS**	Right asymmetry	4 (3.9)
	Left asymmetry	6 (5.9)
	Symmetric	92 (90.2)
**MSL–IC**	Right asymmetry	6 (5.9)
	Left asymmetry	1 (1.0)
	Symmetric	95 (93.1)
**MSL–TR**	Right asymmetry	3 (2.9)
	Left asymmetry	1 (1.0)
	Symmetric	98 (96.1)
**MSL–TI**	Right asymmetry	7 (6.9)
	Left asymmetry	4 (3.9)
	Symmetric	91 (89.2)
**MSL–SPH**	Right asymmetry	1 (1.0)
	Left asymmetry	2 (2.0)
	Symmetric	99 (97.1)

**Table 11:** Distribution of right–left body asymmetries in the vertical plane.

Landmark	Asymmetry Direction	n	%
**A**	Right asymmetry	22	21.6
	Left asymmetry	34	33.3
	Symmetric	46	45.1
**SS**	Right asymmetry	6	5.9
	Left asymmetry	4	3.9
	Symmetric	92	90.2
**IC**	Right asymmetry	1	1.0
	Left asymmetry	3	2.9
	Symmetric	98	96.1
**TR**	Right asymmetry	1	1.0
	Symmetric	101	99.0

**Table 12:** Distribution of patients with head inclinations in the horizontal and vertical planes.

Plane	Parameter	Inclination	n (%)
**Horizontal**	Head position relative to body MSL	Right tilt	22 (21.6)
		Left tilt	34 (33.3)
		No tilt	46 (45.1)
**Vertical**	Body reference line–T	Anterior inclination	62 (60.8)
		No inclination	40 (39.2)

**Table 13:** Distribution of paired right–left and global body asymmetries.

Type of Body Asymmetry	Status	Right Side (n)	Right Side (%)	Left Side (n)	Left Side (%)
**Paired body asymmetries (horizontal plane)**	Absent	68	66.7	66	64.7
	Present	34	33.3	36	35.3
**Paired body asymmetries (vertical plane)**	Absent	80	78.4	66	64.7
	Present	22	21.6	36	35.3
**Global body asymmetries**	Absent	58	56.9	42	41.2
	Present	44	43.1	60	58.8

**Table 14:** Association between paired right–left and global body asymmetries and age group, BMI, and postural body attitude (Pearson’s chi-square test).

Type of Body Asymmetry	Variable	Category	Absent n (%)	Present n (%)	χ^2^	*p* Value
**Paired asymmetries (left side, vertical plane)**	Age group	18–30 years	47 (75.8)	15 (24.2)	8.537	0.014 *
		>30 years	19 (47.5)	21 (52.5)		
**Paired asymmetries (left side, horizontal plane)**	BMI	Underweight	6 (66.7)	3 (33.3)	24.264	<0.001 **
		Normal weight	52 (73.2)	19 (26.8)		
		Overweight	5 (41.7)	7 (58.3)		
		Obese	3 (30.0)	7 (70.0)		
**Paired asymmetries (left side, vertical plane)**	BMI	Underweight	7 (77.8)	2 (22.2)	16.936	0.010 *
		Normal weight	48 (67.6)	23 (32.4)		
		Overweight	7 (58.3)	5 (41.7)		
		Obese	4 (40.0)	6 (60.0)		
**Paired asymmetries (left side, horizontal plane)**	Postural body attitude	Kyphotic	46 (75.4)	15 (34.6)	17.928	0.001 **
		Lordotic	11 (47.8)	12 (52.2)		
		Optimal	9 (50.0)	9 (50.0)		

Values are presented as *n* (% within category). Pearson’s chi-square test (χ^2^). * Statistically significant at *p* ≤ 0.05. ** Highly statistically significant when *p* ≤ 0.01.

**Table 15:** Statistically significant correlations between facial asymmetries.

Facial Asymmetries	Spearman’s ρ	*p*-Value	95% Confidence Interval
**Paired right-sided asymmetries (horizontal plane) vs. paired left-sided asymmetries (horizontal plane)**	−0.538	<0.001 **	−0.666–−0.379
**Global right-sided asymmetries vs. global left-sided asymmetries**	−0.431	<0.001 **	−0.581–−0.253

Spearman’s rank correlation coefficient (ρ): positive correlation when ρ > 0; negative correlation when ρ < 0; negligible correlation when ρ ≈ 0; weak correlation when 0.1 ≤ ρ < 0.3; moderate correlation when 0.3 ≤ ρ < 0.5; strong correlation when ρ ≥ 0.5. ** Highly statistically significant at *p* ≤ 0.01.

**Table 16:** Statistically significant correlations between patients’ postural body asymmetries.

Postural Body Asymmetries	Spearman’s ρ	*p*-Value	95% Confidence Interval
**Paired right-sided asymmetries (horizontal plane) vs. paired left-sided asymmetries (vertical plane)**	0.288	0.003 **	0.093–0.462
**Paired right-sided asymmetries (vertical plane) vs. paired left-sided asymmetries (vertical plane)**	−0.337	<0.001 **	−0.503–−0.147

Spearman’s rank correlation coefficient (ρ) was used to assess associations between ordinal variables. Positive values indicate direct correlations, while negative values indicate inverse correlations. Correlation strength was interpreted as follows: weak (0.1 ≤ ρ < 0.3), moderate (0.3 ≤ ρ < 0.5), and strong (ρ ≥ 0.5). ** Highly statistically significant at *p* ≤ 0.01.

**Table 17:** Statistically significant correlations between facial asymmetries and postural body asymmetries.

Facial Asymmetries	Postural Body Asymmetries	Spearman’s ρ	*p*-Value	95% CI (Lower–Upper)
**Paired, right side, horizontal plane**	Paired, right side, vertical plane	0.229	0.021 *	0.030–0.410
**Global, right side**	Global, right side	0.197	0.047 *	−0.003–0.382

Spearman’s rank correlation coefficient (ρ): positive correlation when ρ > 0; negative correlation when ρ < 0; no correlation when ρ ≈ 0; weak correlation when 0.1 ≤ ρ < 0.3; moderate correlation when 0.3 ≤ ρ < 0.5; strong correlation when ρ ≥ 0.5. * Statistically significant at *p* ≤ 0.05.

## Data Availability

All the data used in this study are available on request from the corresponding author.
